# Following cardiac surgery, do digital drainage systems versus underwater seal impact postoperative outcomes?

**DOI:** 10.1093/icvts/ivaf053

**Published:** 2025-05-02

**Authors:** Alexander Smith, Akshay Patel, Muhammad Mansoor, Raya Almaraihah, Kevin Sales, Khine Wai, Krishna Mani, Adnan Charaf, Marjan Jahangiri

**Affiliations:** Department of Cardiac Surgery, St George’s Hospital, London, UK; Department of Cardiac Surgery, St George’s Hospital, London, UK; Department of Cardiac Surgery, St George’s Hospital, London, UK; Department of Cardiac Surgery, St George’s Hospital, London, UK; Department of Cardiac Surgery, St George’s Hospital, London, UK; Department of Cardiac Surgery, St George’s Hospital, London, UK; Department of Cardiac Surgery, St George’s Hospital, London, UK; Department of Cardiac Surgery, St George’s Hospital, London, UK; Department of Cardiac Surgery, St George’s Hospital, London, UK

**Keywords:** cardiac surgery, digital drain, underwater seal, atrial fibrillation

## Abstract

**OBJECTIVES:**

In this study, we compare digital and underwater seal drainage systems following cardiac surgery and assess postoperative outcomes.

**METHODS:**

Between August 2017 and August 2018, cardiac surgical patients at our hospital were managed postoperatively using underwater seal drainage systems, and between August 2022 and August 2023 using digital drainage systems. Propensity score matching was used to estimate the effect of drainage system on various postoperative parameters (continuous and binary outcome modelling). Primary outcomes were postoperative atrial fibrillation, reoperation for bleeding or tamponade and pleural effusion requiring intervention. Secondary outcomes were hourly and cumulative drain output within 24 postoperative hours.

**RESULTS:**

347 patients met the entry criteria for the study. One hundred ninety patients were managed using an underwater seal drainage system, and 157 patients were managed using a digital drainage system. Three hundred fourteen patients from the original 333 patient cohort were matched according to the drainage system used. After matching, the odds of developing postoperative atrial fibrillation were 0.57 (95% CI 0.32–0.99, *P* = 0.046) times lower in the digital drainage group. There was no difference in the rates of reoperation for bleeding or tamponade, pleural effusion requiring intervention or cumulative drain volume within 24 h.

**CONCLUSIONS:**

In this analysis, the odds of developing postoperative atrial fibrillation were lower in patients managed with digital drainage devices than underwater seal. However, there was no difference in rates of reoperation for bleeding, tamponade, pleural effusion, drain duration or overall length of stay. Digital drainage systems could therefore be considered as part of an enhanced recovery after cardiac surgery pathway.

## Graphical abstract

**Figure:**
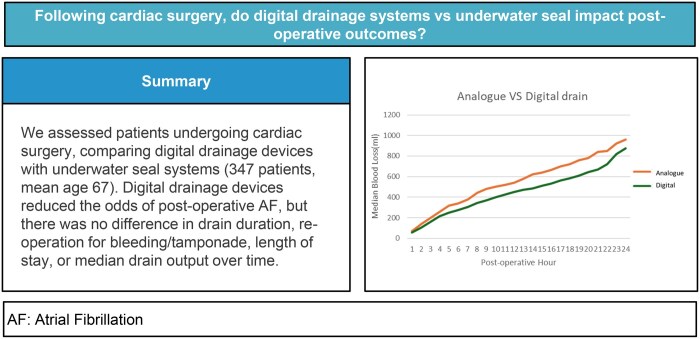

## INTRODUCTION

There is wide variation and lack of consensus on the management of chest tubes after cardiac surgery. Evidence and guidelines regarding the management of drainage systems are limited, and there is significant variance in practice between institutions and surgeons [1]. The advent of digital drainage devices within thoracic surgery, monitoring both fluid and air output, has enabled identification of patients suitable for safe early postoperative drain removal [2–4]. Digital drainage devices provide several advantages over the traditional analogue devices, including the allowance for continuous air leak recordings, provision of an objective manner of judgement as to when to remove a chest tube, maintenance of a stable effective intrathoracic pressure and more [5, 6].

We aim to assess the clinical impact of digital drainage systems after cardiac surgery at a single institution. We set out to compare the rates of postoperative atrial fibrillation (AF), retained blood products (reoperation, tamponade, pleural effusion) and the cumulative drainage within the first 24 h by type of drainage system used (traditional underwater seal ‘Analogue’ versus Medela Thopaz ‘digital’ drain).

## PATIENTS AND METHODS

### Ethical statement

Local audit committee approval for the study was granted. Institutional review board and ethical approval were waived. All data were stored according to local and national guidelines.

Between August 2017 and August 2018, cardiac surgical patients at our hospital were managed postoperatively using underwater seal drainage systems, and between August 2022 and August 2023 using digital drainage systems. Digital devices were introduced in April 2022, and subsequently all cardiac surgical patients were managed using digital devices only. Due to institutional factors and the COVID 19 pandemic, operative numbers were lower from 2019 to 2021, and therefore the period of August 2017–2018 was selected as the comparison group to maximize case numbers. The study included all patients older than 16 years undergoing cardiac surgery under a single surgeon. Patients aged <16 years were excluded.

All patients were managed using a mediastinal drain, with right/left pleural drains if the pleural cavity was breached. Postoperatively, drains were managed on suction −5 Kilopascals and removed following two consecutive hours of no drainage. There was no change in surgical technique over the two time periods, and a posterior pericardiotomy was not performed. Patients received prophylaxis for AF using low-dose bisoprolol on postoperative day 1 unless contraindicated (e.g. bradycardia/severe asthma). All patients had continuous cardiac monitoring postoperatively. A diagnosis of postoperative AF was defined as any new episode of AF whilst an inpatient.

Data were analysed retrospectively using an institutional database to compare the rate of postoperative AF, reoperation for bleeding, tamponade and pleural effusion requiring intervention. Secondary outcomes were hourly and cumulative drain output within the first 24 postoperative hours and total duration of drainage.

### Statistical analysis

Continuous data are reported as mean with standard deviation or median with interquartile range as appropriate to the data distribution, and categorical data as counts and percentages. Continuous data were assessed graphically using histograms and normal–normal plots to judge the data distribution and subsequently compared using the student’s *t*-test or Wilcoxon rank-sum test as appropriate. Univariate analysis between categorical data were conducted using the Pearson Chi-Squared test or Fisher’s exact test in cases where there were less than *n* = 5 in a subgroup.

Propensity score matching was used to estimate the effect of the drainage system on various postoperative parameters (continuous and binary outcome modelling). The propensity scores were estimated using logistic regression based on age, sex, body mass index, use of antiplatelet agents, operative urgency, procedure undertaken, preoperative ejection fraction, cardiopulmonary bypass time and preoperative heart rhythm. One-to-one nearest neighbour matching was used. Three hundred fourteen patients from the original 347 patient cohort were matched according to the drainage system used (digital or underwater seal). A good balance was achieved between the groups, with all standardized mean differences for squares, and two-way interactions between covariates were below 0.15, indicating adequate balance. Calliper matching (0.2 times the standard deviation of the logit of the propensity score) was employed as per Austin [7]. Supplementary Fig. 1 shows the balance of covariates before and after matching. All patients within the range of propensity scores where both treated and control subjects exist were included in the analysis.

Logistic and linear regression modelling were employed according to data distribution of the outcome variable with drain type as the exposure, along with covariates and their interaction as predictors. We included full matching weights in the estimation. The comparisons () function in the *marginaleffects* package was used to perform g-computation in the matched sample to estimate the average treatment effect of the treated population. A cluster-robust variance was used to estimate its standard error with matching stratum membership as the clustering variable.

Hourly median drain output was obtained for the first 24 postoperative hours. Cumulative frequency plots of drain volume were used to determine total output to ascertain the characteristics of total drainage with time. Cumulative drain outputs were compared using a longitudinal mixed-effects regression model considering between- and within-patient, and between drainage system variability, adjusting for the surgical procedure undertaken. All analyses were conducted using R 4.2.3.

## RESULTS

Three hundred forty-seven patients met the entry criteria for the study. One hundred ninety patients were managed using an underwater seal drainage system, and 157 patients were managed using a digital drainage system. The mean age was 67 (+12) years, and 91 (26%) were female. Cardiopulmonary bypass was used in all cases. Median cardiopulmonary bypass time was 72 (61–90) min. One hundred eighty-three (53%) underwent elective, 151 (43%) urgent and 13 (4%) emergency surgery. Urgent surgery was defined as inpatient surgical intervention required following an emergency admission to the hospital, and emergency surgery as immediate unplanned surgical intervention.

Baseline unadjusted characteristics are shown in Table 1. Treatment groups were generally well matched; however, more patients in the digital drainage group required urgent/emergency surgery (digital 88 (56%) versus underwater seal 76 (40%), *P* = 0.02), and more patients in the digital drainage group were receiving preoperative dialysis (digital 10 [6%] versus analogue 3 [2%], *P* = 0.02). Operative characteristics are shown in Table 2.

**Table 1:** Baseline characteristics by treatment group

	Analogue, *n* = 190	Digital, *n* = 157	*P*-value
Age (years)	67 (±12)	67 (±11)	0.65
Gender			
Male	137 (72%)	121 (77%)	
Female	53 (28%)	38 (23%)	0.52
BMI (Kg/m^2^)	29 (±5)	28 (±5)	0.28
LVEF category			
>50%	143 (75%)	123 (78%)	
31–50%	43 (23%)	29 (19%)	
21–30%	2 (1%)	5 (3%)	
<21%	2 (1%)	0 (0%)	0.4
Smoking status			
Current	26 (13%)	15 (10%)	
Ex	80 (42%)	66 (42%)	
Never	84 (45%)	76 (48%)	0.45
Diabetes	53 (28%)	45 (29%)	0.87
Hypertension	117 (62%)	96 (61%)	0.93
Peripheral vascular disease	15 (8)	5 (3%)	0.06
Preoperative dialysis	3 (2%)	10 (6%)	0.02
Redo surgery	6 (3%)	5 (3%)	0.47
Previous AF	14 (7%)	9 (6%)	0.54

AF: atrial fibrillation; BMI: body mass index; LVEF: left ventricular ejection fraction.

**Table 2:** Operative characteristics by treatment group

	Analogue, *n* = 190	Digital, *n* = 157	*P*-value
Preoperative anti-platelet			
Aspirin	112 (59%)	102 (65%)	0.25
Ticagrelor	15 (8%)	15 (10%)	0.58
Clopidogrel	36 (19%)	27 (17%)	0.67
Euroscore II	1.82% (1–3.35)	2.02% (1.2–3.32)	0.28
Urgency			
Elective	114 (60%)	70 (45%)	
Urgent	69 (36%)	81 (51%)	0.01
Emergency	7 (4%)	6 (4%)	
Procedure			
CABG	94 (50%)	81 (51.5%)	
CABG+other	5 (2%)	5 (3%)	
CABG + valve	28 (15%)	17 (11%)	
CABG+valve+other	4 (2%)	4 (3%)	
Major aortic	11 (6%)	15 (9.5%)	
Major aortic+other	0 (0%)	3 (2%)	
Valve alone	27 (14%)	20 (13%)	
Valve + other	21 (11%)	9 (5.5%)	
Other	0 (0%)	3 (1.5%)	0.11
Mini sternotomy	15 (8%)	7 (5%)	0.28
CPB time (min)	70 (59–89)	76 (63–91)	0.34
Cross clamp time (min)	49 (37–68)	47 (38–68)	0.73

CABG: coronary artery bypass grafting; CPB: cardiopulmonary bypass.

Unmatched postoperative outcomes and univariable analysis are shown in Table 3. Underwater seal drainage systems were associated with increased rates of postoperative AF (underwater seal *n* = 50 [26.3%] versus digital *n* = 26 [16.5%], *P* = 0.04).

**Table 3:** Postoperative outcomes by treatment group (unmatched, univariable)

	Analogue, *n* = 190	Digital, *n* = 157	*P*-value
Urgent reoperation	5 (2.6%)	5 (3.1%)	0.95
Tamponade	2 (1%)	3 (1.9%)	0.50
Bleeding	3 (1.6%)	2 (1.3%)	0.81
Pleural effusion requiring drainage	3 (1.6%)	6 (4%)	0.18
Drain duration (hours)	13 (10–19)	12 (10–17)	0.51
Postoperative atrial fibrillation	50 (26.3%)	26 (16.5%)	0.03
Inpatient death	0 (0%)	3 (2%)	0.06
Length of stay (days)	6 (5–8)	6 (5–10)	0.34

Propensity matching showed that after matching for covariates, the odds of developing postoperative AF were 0.57 (95% CI 0.32–0.9, *P* = 0.046) times lower in the digital group than the underwater seal. There was no statistically significant difference in the rates of reoperation for bleeding or cardiac tamponade, pleural effusion requiring drainage. Overall drain duration was 12 (10–17) h in the digital and 13 (10–19) in the underwater seal group (*P* = 0.51). There was no difference in the overall length of stay (*P* = 0.34). Table 4 details the full results of the propensity-matched analysis. There were three (1%) postoperative deaths in the digital drainage group and 0 (0%) in the underwater seal.

**Table 4:** Propensity-matched analysis (*n* = 314)

	Impact of digital drainage system	*P*-value
Incidence of postoperative AF	OR 0.564 (95% CI 0.321–0.991)	0.046
Re-operation	OR 2.4 (95% CI 0.594–9.66)	0.219
Pleural effusion requiring intervention	OR 2.1 (95% CI 0.612–7.82)	0.378
Drain duration (days)	−0.756 days (95% CI −4.13 to 2.61)	0.66
Length of stay (days)	+1.69 days (95% CI −0.139 to 3.53)	0.07

95% CI: 95% confidence interval; OR: odds ratio.

**Figure 1:**
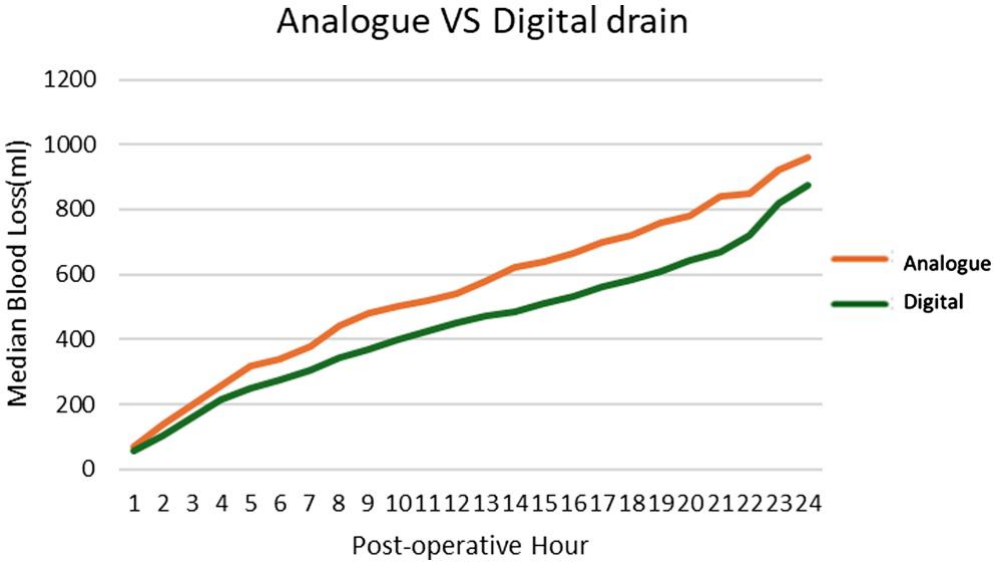
Cumulative median drain output by hour

Figure 1 shows the cumulative median drain output over the first 24 postoperative hours. A longitudinal mixed effects regression analysis was undertaken, considering between- and within-patient, and between drainage system variability adjusting for the type of procedure undertaken. This showed no statistically significant difference in cumulative drainage over 24 h (*P* = 0.09).

## DISCUSSION

The overall results of this study support the safety and efficacy of the digital drainage system, in addition to a reduction in postoperative AF from 26.3% in the underwater seal group to 16.5% in the digital. After propensity matching for covariates, the odds of developing postoperative AF were 0.57 (95% CI 0.32–0.99) times lower in patients managed with digital drainage systems than underwater seal. Other postoperative outcomes did not differ significantly between the treatment groups.

A similar reduction in postoperative AF was found by Kalisnik *et al.* [8] in a prospective cohort study of 1042 patients undergoing elective coronary artery bypass with or without aortic or mitral intervention. They reported that after adjusting for co-variates, traditional underwater seal devices were associated with 2.1 (95% CI 1.4–2.9, *P* < 0.001) times the odds of AF.

The mechanism behind the reduction in AF is unclear. Digital drainage systems provide enhanced regulated suction, with constant monitoring of intrapleural pressures and regulation of suction levels to maintain a constant preselected negative pressure [9]. Kalisnik *et al.* [8] proposed that more efficient clearance of blood postoperatively may reduce rates of pericardial effusion and therefore arrhythmia [10]. Saha *et al.* [11] showed in a retrospective cohort study that the use of digital drainage devices increased blood clearance by 25% within the first six postoperative hours. Similarly, in a small, randomized study, Barozzi *et al.* [12] showed the digital system had enhanced clearance of blood in the early postoperative stages. It is possible, therefore, that enhanced suction and improved clearance of blood via digital drainage systems could reduce rates of pericardial effusion and AF. In our study, however, we found no statistically significant difference in the cumulative postoperative drainage volume. Increased intracavitary suction levels may contribute to the observed reduction in arrhythmia; however, further research is needed to clarify this mechanism.

A small, randomized control study conducted in Germany has previously reported a decrease in the number of drainage-associated complications in cases of re-thoracotomies for tamponade or bleeding, as well as reduced overall chest drainage duration in patients using digital drains [13]. In our study, however, we did not find any significant difference in the rates of reoperation, bleeding, cardiac tamponade or pleural effusion requiring drainage.

### Limitations

Our study is limited by its retrospective single-centre nature, and the use of two different time frames for the experimental and control groups may introduce a calendar time bias. Low numbers of patients required reoperation for bleeding or tamponade, limiting statistical analysis of this outcome. Whilst statistical significance was reached, suggesting lower rates of postoperative AF in the digital drainage group, the evidence was weak with a *P*-value of 0.046. Increased patient numbers and further prospective work are required to confirm our findings.

## CONCLUSION

In this analysis, after propensity matching for covariates, the odds of developing postoperative AF were lower in patients managed with digital drainage devices than with underwater seal. However, there was no difference in rates of reoperation for bleeding, tamponade, pleural effusion, drain duration or overall length of stay. Digital drainage systems could, therefore, be considered as part of an enhanced recovery after cardiac surgery pathway.

## Supplementary Material

ivaf053_Supplementary_Data

## Data Availability

The data underlying the article will be shared on reasonable request to the corresponding author.

## ABBREVIATION

AF: Atrial fibrillation
