# Supplementary figures and images for: Following cardiac surgery, do digital drainage systems versus underwater seal impact postoperative outcomes?
Source: Interdiscip Cardiovasc Thorac Surg. 2025 May 2;40(5):ivaf053. doi: 10.1093/icvts/ivaf053 (PMC12055756; doi:10.1093/icvts/ivaf053)

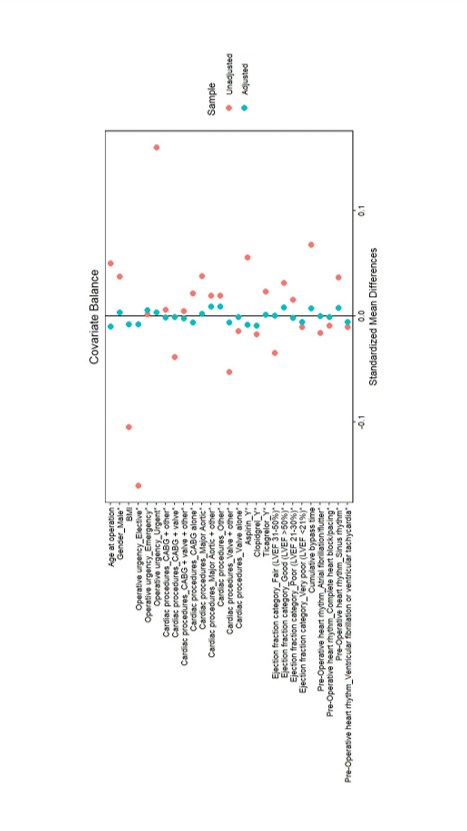

Supplement: ivaf053_Supplementary_Data [file ivaf053_supplementary_data.jpeg]
